# Effects of Elevated Temperature and High and Low Rainfall on the Germination and Growth of the Invasive Alien Plant *Acacia mearnsii*

**DOI:** 10.3390/plants11192633

**Published:** 2022-10-07

**Authors:** Tshililo Kharivha, Sheunesu Ruwanza, Gladman Thondhlana

**Affiliations:** 1 Department of Environmental Science, Rhodes University, Makhanda 6140, South Africa; 2 Department of Environmental Science and Centre of Excellence for Invasion Biology, Rhodes University, Makhanda 6140, South Africa

**Keywords:** global change, biological invasions, warming, soil drought, plant response

## Abstract

The impact of climate change on the germination and growth of invasive alien plants varies depending on the plant species and invasion process. We experimentally assessed the responses of the invasive alien plant *Acacia mearnsii* to future climate change scenarios—namely, elevated temperature as well as high and low rainfall. *Acacia mearnsii* was grown at an elevated air temperature (+2 °C), high rainfall (6 mm per day), and low rainfall (1.5 mm per day), and its germination and growth performance were measured over five months. We further examined changes in soil nutrients to assess if the above-mentioned climate change scenarios affected soils. Both elevated temperature and high rainfall did not influence *A. mearnsii* germination and seedling growth. In contrast, we observed reductions in *A. mearnsii* germination and growth in the low rainfall treatment, an indication that future drought conditions might negatively affect *A. mearnsii* invasion. We noted that elevated temperature and rainfall resulted in varied effects on soil properties (particularly soil C, N, Ca, and Mg content). We conclude that both elevated temperature and high rainfall may not enhance *A. mearnsii* invasion through altering germination and growth, but a decrease in *A. mearnsii* invasiveness is possible under low rainfall conditions.

## 1. Introduction

Invasion by invasive alien plants is considered one of the major drivers of biodiversity loss in South Africa [1,2]. The impacts of major plant invaders such as *Acacias*, *Pines*, *Lantana*, and *Eucalyptus* species on South African ecosystems are huge and include loss of water [3], displacement of native endemic species [1], and loss of productive land (e.g., rangelands) [4]. Invasion by invasive alien plants is estimated to cost South Africa billions of rands in economic losses mainly through water, rangeland, and biodiversity loss [3,5,6]. From a management standpoint, South Africa spent more than ZAR 2 billion per year (approximately USD 111 million at the current exchange rate), investing in the control of invasive alien plants through the national Working for Water (WfW) programme [2,3]. The negative socio-economic and environmental impacts posed by invasive alien plants are expected to increase due to several factors such as climate change, increased international travel and trade, as well as habitat destruction [2]. Climate change is expected to exacerbate the extent and impacts of most invasive alien plants through range expansion and increase in plant density [7]. However, it remains unclear how individual invasive alien plant species will respond to climate change, as mixed results on plant germination and growth have been reported under various climate change scenarios [7,8].

Predicting how invasive alien plants will respond to future climate change scenarios is important given the huge adverse socio-economic and ecological effects of invasive alien plants [9,10]. The recent Intergovernmental Panel on Climate Change (IPCC) report suggests that global warming will increase due to an increase in greenhouse gases; however, temperature increases are expected to be greater in winter than in summer [7,11]. Future changes in rainfall patterns are predicted, but these are expected to vary, with significant drying in some regions and increases in rainfall frequency and intensity in other areas [7,11]. South Africa is expected to experience a reduction in precipitation, increased droughts, and a general increase in temperature ranging from 1.5 °C to 2 °C depending on region [11]. These changes in temperature and rainfall are expected to modify the distribution of plant species in general. From an invasive alien plant standpoint, it is generally predicted that future climatic changes will aid alien plant invasion, creating new habitats and niches for expansion [7,12]. However, the few experimental studies on individual species’ responses to manipulated temperature and rainfall show mixed results [7]. For example, Dukes [13] and Dukes et al. [14] reported that the invasive herb species *Centaurea solstitialis* L. responds positively to future increases in CO_2_ and soil nutrients, but negatively to increases in precipitation. Chuine et al. [15] reported that artificial warming enhanced the growth and biomass of the invasive grass *Setaria parviflora* as compared to the native grasses. Verlinden et al. [16] observed that warming enhanced the aboveground biomass of the invasive herb species *Senecio inaequidens*, but it did not modify its competitive interaction with the native competitor *Plantago lanceolata*. Haeuser et al. [17] reported that nine alien herbs were negatively affected by reduced watering but had better growth performance compared to native herbs under increased temperature. In all these experiments that mimic climate change, invasive alien plant species’ responses were linked to individual plant’s (i) adaptability, (ii) plasticity, and (iii) physiological responses to climate change [16]. Overall, climate change experimental studies seem to indicate that individual invasive alien plant species respond differently to climate change, an indication that changes are species-dependent.

It is possible that plant responses to future climate change scenarios, particularly temperature and rainfall, are linked to changes in soil properties. However, very few studies have looked at how soil properties will change underneath different invasive alien plant species in response to climate change [7]. For example, the *Centaurea solstitialis* growth response to temperature change was perceived to be dependent on changes in soil moisture [14]. Both increases in future climatic changes in temperature and rainfall are expected to influence soil nutrients, particularly soil N and C, with some studies suggesting an increase due to increased future plant growth (implying more litter) and CO_2_ enrichment [18]. Other studies suggest a decrease due to nutrient leaching and decreased soil moisture, which will subsequently affect microbial activity [19]. Overall, future changes in soil nutrients will ultimately influence how a plant species responds to future changes in climate. Yet, future changes in soil nutrients are complex to predict given that soil systems are interconnected to other direct and indirect factors such as organic matter, soil decomposition, and land-use patterns [20].

In comparison to distribution models, climate change experiments can provide better insights for understanding the responses of invasive alien plants to climate change [16]. The challenges associated with the use of distribution models to predict plant species’ responses to climate change are: (i) data availability, (ii) methodological difficulties, (iii) model selection and predictor challenges, and (iv) parameterization and model accuracy [21]. Therefore, the use of experiments to assess future plant changes and responses to climate change presents a better method since it is observational. However, most experimental work is conducted under microcosm conditions or small-scale field-based experiments, thus, potentially affecting the accuracy of results, relevancy to community ecology, and challenges with policy applicability [22]. Although climate change experimental studies are uncommon due to the above-mentioned challenges that minimize the ability to generalize results, various studies have shown that experimental studies provide useful information that can advance our understanding of climate change effects on plants [7,22]. However, most experimental studies have focused on the performance of native plants following CO_2_ and temperature manipulations, with a few notable exceptions on invasive alien plant species, particularly on temperature and rainfall change [13,16]. In addition, the above-mentioned few experimental studies are mostly on alien herbs and grasses [8,13,14], as compared to alien trees and shrubs which are understudied. To effectively manage invasive alien plants in future, more experimental studies are needed to predict how individual plant species respond in future climatic changes, and to allow conclusive judgements. Both temperature and rainfall may influence the germination, growth, distribution, and reproductive success of invasive alien plants, thus, the need to conduct species-specific experimental studies [7,16]. Here, we tested experimentally how a well-known problematic invasive alien tree in South Africa—namely, *A. mearnsii*—will respond to elevated temperature as well as high and low rainfall. Examining these climate change factors individually is important for examining the direct effects on the plant.

*Acacia mearnsii*, commonly known as black wattle, is a tree species belonging to the Fabaceae family, and was introduced to South Africa from Australia in the 1850s for bark and wood plantation purposes [2]. Anecdotal reports suggest that *A. mearnsii* invasion covers more than 440,000 ha of the country’s land and is increasing [23]. *Acacia mearnsii* is regarded as a prolific invader that dominates the mesic regions of the country, particularly the Eastern Cape, KwaZulu-Natal, and Mpumalanga Provinces [24]. Although *A. mearnsii* has several socio-economic benefits such as timber and firewood, the plant species is regarded as an ecosystem transformer with several negative impacts, such as the displacement of native species, reduction in water supply, soil manipulation (particularly Nitrogen fixation), and loss of grazing land [23,25]. It is perceived that the costs of *A. mearnsii* invasion outweigh the benefits associated with the species, and most costs are associated with control initiatives [23]. Various control options, such as biocontrol [26] and mechanical clearing have been introduced to manage *A. mearnsii* invasion; however, the results are rather mixed [23], with management challenges being linked to a persistent soil seed bank which facilitates reinvasion [23,25].

In this study, we aimed to experimentally assess the response of *A. mearnsii,* a prolific alien invader, to climate change scenarios in South Africa. More precisely, we aimed to investigate how the plant responds to individual changes of increased temperature (+2 °C) as well as increased and reduced rainfall. After the experiment, we further examined changes in soil nutrients to assess if the above-mentioned climate change scenarios affect soil properties. Key research questions included: (i) how does increased temperature and rainfall, as well as reduced rainfall, affect the performance (germination and growth) of *A. mearnsii,* and (ii) how does increased temperature and rainfall, as well as reduced rainfall, affect soil nutrients underneath *A. mearnsii* seedlings. Given the experimental approach used in this study, results of this study can help to investigate the effects of climate change (temperature and rainfall) on *A. mearnsii*, thus, providing valuable insight needed to manage the invasive alien plant.

## 2. Results

### 2.1. Effects of Temperature and Rainfall on Acacia mearnsii Germination and Growth

**Figure 1:**
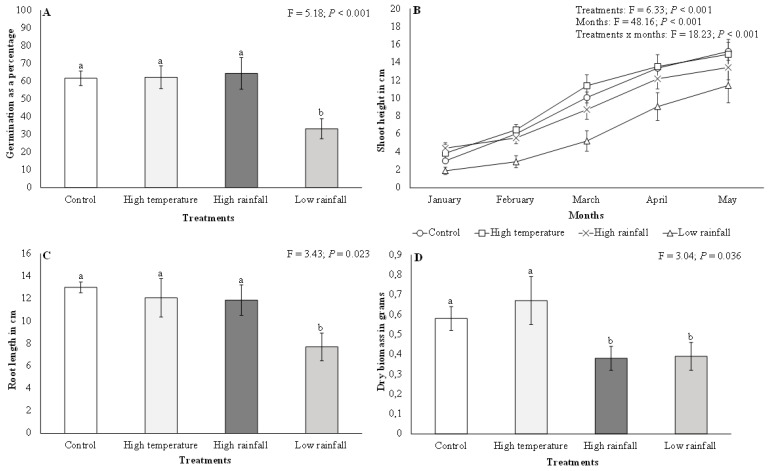
Effects of elevated temperature and high and low rainfall on *Acacia mearnsii* (**A**) germination as a percentage, (**B**) shoot height in cm, (**C**) root length in cm, and (**D**) dry biomass in grams. Bars are means ± SE and ANOVA results are shown. Bars with different letter superscripts are significantly different.

There were significant (F = 5.18; *p* < 0.001) differences in *A. mearnsii* germination among the different treatments. *Acacia mearnsii* germination was twice as high in the high temperature (mean = 62.27% ± 6.44 SE), high rainfall (mean = 64.40% ± 8.91 SE), and control (mean = 61.65% ± 4.08 SE) treatments than in the low rainfall (mean = 33.20% ± 5.66 SE) treatment (Figure 1A). There were significant (F = 6.33; *p* < 0.001) differences in *A. mearnsii* shoot height among the different treatments (Figure 1B). Similarly, monthly comparisons showed significant (F = 48.16; *p* < 0.001) differences for *A. mearnsii* shoot height. Shoot height in all treatments increased between January and May (Figure 1B), with the highest average shoot height growth rate being recorded in the high temperature (11 cm) and control (12 cm) treatments as compared to the high and low rainfall treatments (9 cm, respectively). Interactions between treatments and months for *A. mearnsii* shoot height showed significant (F = 18.23; *p* < 0.001) differences (Figure 1B). There were significant (F = 3.43; *p* < 0.01) differences in *A. mearnsii* root length among the different treatments (Figure 1C). A Tukey test indicated that *A. mearnsii* root length was highest in the high temperature (mean = 12.07 cm ± 1.71 SE), high rainfall (mean = 11.87 cm ± 1.36 SE), and control (mean = 13.00 cm ± 0.48 SE) treatments as compared to the low rainfall (mean = 7.70 cm ± 1.23 SE) treatment.

**Figure 2:**
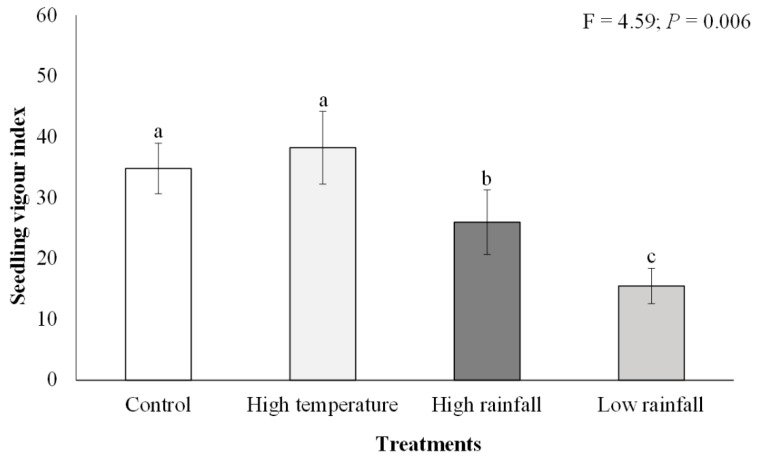
Effects of elevated temperature and high and low rainfall on *Acacia mearnsii* seedling vigour index II. Bars are means ± SE and one-way ANOVA results are shown. Bars with different letter superscripts are significantly different.

Dry biomass was significantly higher (F = 3.04; *p* < 0.05) in the high temperature (mean = 0.67 g ± 0.12 SE) and control (mean = 0.58 g ± 0.06 SE) treatments than in the high (mean = 0.38 g ± 0.06 SE) and low rainfall (mean = 0.39 g ± 0.07 SE) treatments, which had the lowest dry biomass (Figure 1D). There were significant differences (F = 4.59; *p* < 0.01) in *A. mearnsii* seedling vigour among the different treatments (Figure 2). *Acacia mearnsii* seedling vigour was highest in the high temperature and control treatments as compared to the high and low rainfall treatments. Seedling vigour was twice as high in the high temperature (mean = 38.23 ± 5.98) and control (mean = 34.79 ± 4.16) treatment compared to the low rainfall treatment (mean = 15.48 ± 2.92).

### 2.2. Effects of Temperature and Rainfall on Soil Properties after Acacia mearnsii Growth

Both soil C and N were significantly (*p* < 0.05) higher in the high temperature treatment (mean = 3.13% ± 0.13 SE and mean = 0.19% ± 0.01 SE, respectively) compared to the low rainfall (mean = 1.85% ± 0.09 SE and mean = 0.10% ± 0.01 SE, respectively) treatment (Table 1). However, a Tukey test showed no significant (*p* > 0.05) differences among the high temperature, high rainfall, and the control treatments for both soil C and N. Similarly, the exchangeable cations of Ca and Mg were significantly (*p* < 0.05) higher in high temperature (mean = 4.57 cmol/kg ± 0.28 SE and mean = 3.13 cmol/kg ± 0.17 SE, respectively) than in the low rainfall (mean = 3.13 cmol/kg ± 0.03 SE and mean = 2.10 cmol/kg ± 0.01 SE, respectively) treatment (Table 1). A Tukey test showed no significant (*p* > 0.05) differences among the high temperature, high rainfall, and the control treatments for both Ca and Mg. Soil pH, P, K, and Na showed no significant (*p* > 0.05) differences among the treatments (Table 1).

## 3. Discussion

This study examined *A. mearnsii* seed germination and growth in response to elevated temperature and high and low rainfall. Although greenhouse-based experimental studies on climate change have been criticized, results of this study allowed the detection of *A. mearnsii* responses to increased temperature and rainfall. Manipulative greenhouse-based experiments have been shown to provide results that can be used to assess climate change effects on plants [7,13,14]. The results show that relative to the control treatment, both increased temperature and rainfall did not influence *A. mearnsii* seedling germination and growth. However, the reported high seedling germination percentage (above 60%) and growth rate (above 9 cm) in the high temperature and rainfall treatments are indications that *A. mearnsii* will adapt and tolerate predicted future temperature and rainfall increases. In contrast, our results also showed reductions in *A. mearnsii* germination and growth rate in the low rainfall treatment, an indication that future decreases in rainfall will negatively affect *A. mearnsii* germination and growth. We also observed that elevated temperature and high and low rainfall will cause varied effects on soil properties underneath the growth of *A. mearnsii*.

Previous studies on invasive alien plant species’ responses to increased temperature have reported mixed results. For example, Chen et al. [8] reported that an increase in temperature did not influence germination and growth on four invasive herbs, namely, *Eupatorium catarium*, *Mikania micrantha*, *Bidens pilosa* var. radiate, and *Ageratum conyzoides*. With specific reference to the above-mentioned four species, Chen et al. [8] concluded that increased warming may not facilitate plant invasion due to warming-related growth suppression. In contrast, Yuan and Wen [27] reported that increased temperature resulted in high germination and seedling growth of three invasive alien herb species of *Crassocephalum crepidioides*, *Conyza canadensis*, and *Ageratum conyzoides*. The above-mentioned study concluded that these invasive plant species can adapt to increased temperature and water stress, an attribute that could make them more invasive in future. Similarly, Zhou and He [28] reported that climatic warming decreased the germination of the invasive alien herb species *Solidago canadensis*. Udo et al. [29] showed that although the invasive alien plant species *Ulex europaeus* has a wide germination temperature range, seed germination tends to decrease at higher temperatures—this is likely to explain its restricted distribution range in tropical environments. Increased temperature has been shown to negatively affect plant germination and growth through several mechanisms. Zhang et al. [21] and Dülger et al. [30] reported that increased temperature causes nutrient dilution in plants, a process where nutrients such as nitrogen become limited in the plant, thus, leading to stunted plant growth. Other studies attribute reduced plant growth under increased temperature to altered plant leaf enzyme function [31,32], increasing transpiration [33], increasing stomatal conductance, and decreasing plant photosynthetic metabolism—of which the latter is known to be sensitive to temperature changes [34]. Lee et al. [35] reported that the increase in temperature negatively affects plant growth through decreases in the leaf area, delay in tuber initiation, and carbon assimilation inhibition. However, some plants can develop mechanisms that allow them to adapt to increased temperature, thus, resulting in enhanced growth. For example, Luo et al. [36] reported that plants with greater root resilience and larger root size can grow better under increased temperature.

Increase in rainfall had no effect on *A. mearnsii* germination and growth. In fact, *A. mearnsii* dry biomass was lower in the high rainfall than in the control treatment. Our results concur with observations by Eskelinen and Harrison [37], who found that the growth rate of invasive alien herb species *Centaurea solstitialis* and grass species *Aegilops triuncialis* was not influenced by rainfall increase, particularly in nutrient-poor habitats. The above-mentioned study concluded that increased rainfall alone does not benefit invasive alien species growth, but synergies among rainfall increase, soil nutrient enhancement, and reduced plant competition are better at explaining future plant invasion under increased rainfall conditions. Indeed, the effects of increased rainfall on plant growth are species- and site-dependent, because, in some cases, increased rainfall can enhance plant growth through stimulating photosynthesis, transpiration, and carbon intake [38]. In contrast, increased rainfall can trigger oxygen deficiency, nutrient loss, and soil crusting which ultimately prevent plant germination and growth [39]. Other studies have reported that increased rainfall increases soil moisture, which subsequently reduces infiltration [40]. Reduced infiltration is associated with poor soil aeration which can lead to nitrogen volatilisation, reduced availability of nutrients to plants, poor root function, and stunted plant growth. Although not measured in this study, it is possible that rainfall increases could have affected *A. mearnsii* germination and growth through soil nutrient loss and reduced nutrient uptake.

Most studies on bioclimatic modelling of invasive alien plants suggest shifts and range expansion for most invasive alien species, although changes could be species-, site-, and biome-dependent [41,42]. Although future climate warming can result in different effects on invasive alien plants, our greenhouse-based experimental results on *A. mearnsii* under increased temperature and rainfall show no significant effects relative to the control treatment. This seems to suggest that increased temperature and rainfall may not increase *A. mearnsii* invasion potential in the future via enhanced germination and seedling growth. Our results provide no evidence to support the generalised hypothesis that increased warming would facilitate plant invasion [7]. This can be explained by the fact that the ability to predict alien plant invasion under future climate change scenarios is complicated given the multiple factors that influence plant invasion, e.g., plant traits, land-use changes, local invasion patterns, and biotic and abiotic interactions [43]. For example, Dukes et al. [14] used manipulated experiments which showed conflicting results, with some species responding positively to elevated carbon dioxide and negatively to temperature and precipitation increase. However, it is important to note that invasive alien plants can adapt rapidly to future environmental changes [44], thus, it is possible for *A. mearnsii* to be more invasive in future. The ability of *A. mearnsii* to adapt rapidly to future climate change implies that it can easily overcome invasion barriers and constraints caused by climate change, thus, becoming more invasive in future [42]. For example, Wu and Yu [45] showed that although soil microorganisms underneath *A. mearnsii* soils are sensitive to increased warming and elevated CO_2_, *A. mearnsii* tends to respond by increasing root exudates and root length, which subsequently increases its growth through increased nutrient uptake and photosynthetic capacity. Based on the above-mentioned study, the positive effect of increased warming and elevated CO_2_ on *A. mearnsii* root exudates and root length can be viewed as an environmental adaptation strategy. However, this environmental adaptation strategy could be species-specific since it was not observed on the invasive alien plant species *Eucalyptus urophylla*, which was subjected to similar warming and elevated CO_2_ conditions [45].

We observed a decrease in *A. mearnsii* germination and growth in the low rainfall treatment. Our result suggests that future invasions by *A. mearnsii* will be reduced under low rainfall scenarios, a result that was observed by Kelso et al. [46] who showed that the densities of the invasive alien herb species *Lepidium latifolium* will be drastically reduced under future drought conditions. Fahey et al. [47] reported that low rainfall reduced the diversity of the invasive grass *Imperata cylindrica* by 20%, but the same study observed no significant effect of low rainfall on the cover of *I. cylindrica*. Recently, Orbán et al. [48] reported that drought suppressed the germination of some invasive species, namely, *Ambrosia artemisiifolia*, *Cenchrus incertus*, and *Asclepias syriaca*. In contrast, Crous et al. [49] showed that the invasive alien species *A. mearnsii* has high drought tolerance levels due to low water potential hydraulic conductivity loss compared to co-occurring native species in the Fynbos region of South Africa. In our study, the response of *A. mearnsii* to low rainfall is linked to how the plant tolerates water stress. Although we did not test this, it is plausible that *A. mearnsii* experienced low germination and growth under insufficient water conditions due to stress, embolism (air bubbles in the xylem that prevent water movement in the plant), and carbon starvation (reduction in plant-stored sugars and starches) [50]. Water stress in plants triggers stomatal closure to decrease water loss via transpiration [51]. However, stomatal closure reduces CO_2_ diffusion in plant leaves, leading to carbon assimilation [52], which will affect carbon balance, carbon starvation, and subsequent hydraulic failure (xylem dysfunctional). The above-mentioned water stress-related challenges are likely to result in reduced plant growth and mortality. Indeed, our results on the dry biomass of *A. mearnsii* confirmed that low rainfall affects plant growth and biomass.

The effects of increased temperature and high and low rainfall on soil properties are mostly driven by changes in soil temperature and moisture [53]. The high temperature and rainfall treatments recorded high soil C, N, Ca, and Mg content as compared to the low rainfall treatment—an indication that the former treatments might trigger positive changes on some soil properties underneath the invasive species, *A. mearnsii*. Some studies have shown that an increase in temperature can result in varied changes in soil properties, e.g., an increase in soil P, pH, and total salts, but not in soil N and P content [54]. Other studies have reported that increased temperature accelerates the mineralization of soil organic carbon, resulting in increased microbial decomposition, thereby causing nutrient accumulation, which is associated with increased soil nutrients [54,55]. However, some studies have reported no changes in some soil properties (particularly soil N and P content) under increased temperature [56]. Under increased temperature conditions, other soil processes and functions will be negatively affected, e.g., poor soil structure, soil stability, and soil water-holding capacity. Future changes in soil processes and functionality will have either positive or negative effects on soil properties; however, variations will depend on the specific nutrient and site [57]. Under increased rainfall conditions, soil N deposition is expected to increase due to flooding and soil disturbance, thus, rendering the amount of soil N to be higher in soils [57]. In contrast, recent studies have reported that high rainfall causes soil nutrient loss through surface runoff which is known to promote the dissolution and leaching of soil nutrients [58]. The effect of low rainfall on soil properties are varied, however, most studies have reported a decrease in soil nutrients under a low rainfall condition because of changes in soil moisture which ultimately affect nutrient decomposition [59]. Additionally, due to reduced plant growth under low rainfall conditions, the release of litter is likely to be low, resulting in consequent changes in soil organic matter and reductions in soil nutrients, particularly soil C and N content [59].

## 4. Materials and Methods

### 4.1. Sampling Site

Soils for the greenhouse-based experiment were collected in early November 2019 from a natural vegetated area located outside Bathurst town (33°30′14″S; 26°49′26″E), approximately 43 km from Makhanda in the Eastern Cape Province of South Africa. The above-mentioned area was selected because of its proximity to dense stands of *A. mearnsii* (10 km apart). Vegetation in the area where the soils were collected is classified as the Albany Coastal Belt, which is within the Albany Thicket Biome [60]. The Albany Coastal Belt is dominated by short grasslands and scattered bush clumps, mainly *Vachellia natalitia*. Due to proximity to the Indian Ocean, temperatures in the area are mild with mean annual temperatures of approximately 17.8 °C [60]. The mean annual precipitation in the area is approximately 677 mm, with most precipitation in the summer months between October and April [60]. Soils are generally sandy, deriving from Beaufort Group mudstone and Sandstone in the northeast Nanaga formation arenite [60].

### 4.2. Soil Collection and Experimental Design

Soil samples were collected along four transects that were 10 m apart using a soil corer measuring 10 cm diameter at 10 cm depth. At each transect, 15 soil cores were collected 5 m apart. Collected soils were sieved using a 2 mm mesh to remove all the debris, rocks, plant litter, and seeds of other plants. After sieving, the soils were sterilized in an oven drier at 120 °C for two days. Soil sterilization aimed to eliminate soil biota. After soil sterilization, 500 g of sterilized soil were transferred into germination pots measuring 10 cm diameter × 10 cm depth. The pots were placed in plastic containers each measuring 1 m long × 45 cm wide × 15 cm high. Each plastic container contained 15 replicated pots per treatment. Plastic containers with pots were transferred to a passively ventilated greenhouse, which allowed air temperature to be close to outside temperature, at Rhodes University.

The experimental design in the greenhouse had four treatments which simulated future temperature and rainfall scenarios. The treatments were high temperature, high rainfall, low rainfall, and control. The high temperature treatment was achieved by suspending one infra-red lamp at 1.2 m from the plastic container with the 15 pots. The high temperature treatment received a downward radiant flux of 175 watts per square meter, supplied by suspending infrared lamps (Philips incandescent PAR38 IR 175R, Philips, Amsterdam, The Netherlands). The infra-red lamp elevated air temperature to +2 °C above ambient temperature (surrounding temperature), which is consistent with future South African temperature change scenarios [11,61]. Soil temperature was calibrated and monitored at +2 °C using data loggers (Higrochron Hi-Res iButtons) that were placed 5 cm in the soil and recorded the soil temperature at hourly intervals. Soil temperature calibration and monitoring were conducted in comparison to the soil temperature recorded in data loggers that were placed in the control treatment (i.e., with no energized infra lamp). The high rainfall treatment was achieved by watering 15 pots in a plastic container three times a week with above-average daily precipitation, thus, each pot received 6 mm per day. In contrast, the low rainfall treatment was achieved by watering 15 pots in a plastic container three times a week with below-average daily precipitation, thus, each pot received 1.5 mm per day. Calculations for both high and low rainfall estimates were carried out using the study area daily average precipitations for 2017 and 2018, which was estimated at 3 mm per day. A control treatment with no above-mentioned temperature and rainfall treatments consisted of 15 pots in a plastic container, and these received the normal watering of 3 mm three times a week. Throughout the experiment, watering was carried out three times a week to avoid waterlogging conditions and to maintain soil moisture, allowing the seedlings to grow and develop.

### 4.3. Germination and Seedling Growth Measurements

In each of the 60 pots (15 pots × four treatments) that were arranged in four plastic containers, three seeds of *A. mearnsii* were sown at a depth of 5–10 mm in late November 2019. After one month, germinating seedlings were counted and later thinned to one per pot; then, seedling growth was monitored for the five summer months (January to May 2020) that are associated with seed germination and growth under natural conditions. Seedling growth rate (shoot height in cm) was measured monthly using a ruler. At the end of the experiment in May, all seedlings were harvested, and their root length and total dry biomass were measured. To measure root length, soils were carefully washed from harvested seedlings and root length was measured using a ruler in centimetres (cm). To measure dry biomass, the harvested seedlings were oven dried at 105 °C for two days and weighed with a scale to determine the total dry biomass in grams (g).

### 4.4. Soil Analysis after Seedling Harvesting

After *A. mearnsii* seedlings were harvested, three soil sample replicates were collected from all the treatments for analysis (12 samples in total). The decision to analyse three replicates per treatment was based on financial limitations and the assumption that there would be no marked soil property variations within all the 15 pots per treatment since they were subjected to similar climate change scenarios. Soil pH was measured in 1:5 soil: KCl extracts, then filtered and analysed by atomic absorption spectrometry (SP428: LECO Corporation). Soil P was analysed using the Bray-II extract method [62]. Total soil C was analysed using the modified Walkley–Black method [63]. Total soil N was analysed by complete combustion using an elemental analyser (Euro EA; Eurovector, Milan, Italy). Soil K, Na, Ca, and Mg content were extracted in 1:10 ammonium acetate solution using the centrifuge procedure. They were filtered and analysed by atomic absorption spectrometry (SP428; LECO Corporation, St. Joseph, MI, USA).

### 4.5. Statistical Analysis

The effect of the various climate change treatments on *A. mearnsii* germination, root length, and dry biomass were compared using a one-way ANOVA since the data were collected once, whereas the effects of the various treatments on shoot height were compared using repeated measures ANOVA since the data were measured monthly for five months. Repeated measures ANOVA was used to determine the changes over both plant growth and months. The germination rate was calculated as the total number of seedlings that germinated per pot in comparison to the total number of seeds planted, expressed as a percentage. Calculations for average shoot height growth rate were based on the first and final shoot readings. Seedling vigour index II, which reflects rapid seedling emergency and plant performance, was calculated based on the method suggested by Kumar et al. [64]. Calculations were based on the equation:

SVI-II = MGP × DBP

where SVI-II is seedling vigour index II, MGP is mean germination per pot, and DBP is dry biomass per pot. All soil properties were analysed using a one-way ANOVA since the data were collected once at the end of the experiment. Assumption of normality and proof of homogeneity of variance were tested using Kolmogorov–Smirnov tests and Levene’s test, respectively. Data were normally distributed. Where ANOVAs were significantly different, the Tukey Honestly Significant Difference (HSD) post hoc test was used at *p* < 0.05. All statistical tests were carried out using TIBCO STATISTICA version 14.0 software [65].

## 5. Conclusions

Our experimental-based results showed that future increases in temperature and rainfall may not significantly alter germination and growth of the invasive alien species *A. mearnsii*, whereas future low rainfall conditions might decrease *A. mearnsii* germination and growth. The effects of elevated temperature as well as high and low rainfall on soil nutrients underneath *A. mearnsii* were varied, with high soil C, N, Ca, and Mg only being observed under a high temperature condition. In South Africa, *A. mearnsii* is a well-known aggressive invader which germinates and grows quickly as compared to native species [25]. Our results are not consistent with global change models that have predicted increases in plant invasion due to future increases in temperature and rainfall [7]. However, we are aware that *A. mearnsii* might increase in the future due to species-specific environmental adaptation strategies and a complex interplay of temperature, rainfall, and other factors. Overall, the results suggest that evaluating alien plant invasion responses to future global change scenario is complex, making it hard to determine future invasion expansion trajectories [8]. Complexities are linked to (i) the temperature and rainfall effects on the individual invasive alien species (e.g., plant growth and physiology), (ii) the indirect effects of temperature and rainfall changes on resources (e.g., water and soil nutrients) and interactions with other plants, and (iii) the temperature and rainfall effects on external factors (e.g., landscape changes and human influence) [8]. Therefore, experiments on the responses of invasive alien species to climate change need to incorporate the above-mentioned complexities to understand future responses.

## Figures and Tables

**Table 1:** Effects of elevated temperature and high and low rainfall on soil properties after *Acacia mearnsii* seedling growth. Data are means ± SE and one-way ANOVA results are shown. Means with different superscript letters show significant differences (*p* ≤ 0.05) among treatments. Values within columns with different letter superscripts are significantly different.

	Control	High Temperature	High Rainfall	Low Rainfall	F-Values	*p*-Values
pH	6.50 ± 0.10 ^a^	6.53 ± 0.09 ^a^	6.50 ± 0.06 ^a^	6.37 ± 0.07 ^a^	0.89	0.502
Total nutrient concentration						
P Bray II (mg/kg)	38.17 ± 27.17 ^a^	52.70 ± 42.65 ^a^	12.27 ± 2.44 ^a^	11.93 ± 1.23 ^a^	0.63	0.614
C (%)	2.72 ± 0.31 ^ab^	3.13 ± 0.13 ^a^	2.25 ± 0.36 ^ab^	1.85 ± 0.09 ^b^	5.03	0.030
N (%)	0.14 ± 0.02 ^ab^	0.19 ± 0.01 ^a^	0.12 ± 0.02 ^ab^	0.10 ± 0.01 ^b^	5.51	0.023
Exchangeable cations (cmol/kg)						
Ca	4.00 ± 0.47 ^ab^	4.57 ± 0.28 ^a^	3.77 ± 0.20 ^ab^	3.13 ± 0.03 ^b^	4.06	0.050
Mg	2.43 ± 0.19 ^ab^	3.13 ± 0.17 ^a^	2.27 ± 0.32 ^ab^	2.10 ± 0.01 ^b^	5.05	0.030
K	0.14 ± 0.01 ^a^	0.17 ± 0.01 ^a^	0.13 ± 0.01 ^a^	0.14 ± 0.01 ^a^	2.35	0.149
Na	0.28 ± 0.03 ^a^	0.39 ± 0.04 ^a^	0.27 ± 0.03 ^a^	0.28 ± 0.02 ^a^	3.78	0.059

## Data Availability

Not applicable.
